# Draft Genome Sequence of *Anoxybacillus suryakundensis* Strain JS1^T^ (DSM 27374^T^) Isolated from a Hot Spring in Jharkhand, India

**DOI:** 10.1128/genomeA.00824-16

**Published:** 2016-08-11

**Authors:** Kamal Deep, Abhijit Poddar, William B. Whitman, Subrata K. Das

**Affiliations:** a Department of Biotechnology, Institute of Life Sciences, Nalco Square, Bhubaneswar, India; b Department of Microbiology, University of Georgia, Athens, Georgia, USA

## Abstract

*Anoxybacillus suryakundensis* strain JS1^T^, a facultative anaerobic, moderately thermophilic, alkalitolerant bacterium, was isolated from a hot spring. The estimated genome is 2.6 Mb and encodes 2,668 proteins.

## GENOME ANNOUNCEMENT

*Anoxybacillus suryakundensis* strain JS1^T^ is a Gram-positive, nonmotile, facultative anaerobe. A moderately thermophilic and alkalitolerant bacterium, it was isolated from sediment samples from a hot spring in Suryakund, Jharkhand, India. Heterotrophic growth occurred at 40 to 60°C and pH 5.5 to 11.5 (1).

The draft genome of *A. suryakundensis* was sequenced using the Illumina HiSeq 2000 platform, which generated 11,125,280 reads totaling 1,679.9 Mb. Filtered Illumina reads were assembled using Velvet version 1.2.07 (2), and 1- to 3-kb simulated paired-end reads were created from Velvet contigs using wgsim version 0.3.0 (https://github.com/lh3/wgsim). Illumina reads were assembled with simulated read pairs using Allpaths-LG version r46652 (3).

The genome was annotated using the JGI Microbial Genome Annotation Pipeline (4). Genes were identified using Prodigal (5), followed by manual curation using GenePRIMP (6) for finished genomes and draft genomes in fewer than 20 scaffolds (>50 kb) containing 92.9% of the genome. The final assembly was based on 1,510.0 Mb of Illumina data with 302-fold input read coverage. The predicted coding sequences (CDSs) were translated and used to search the NCBI nonredundant database and the UniProt, TIGRFam, Pfam, KEGG, COG, and InterPro databases. The tRNAScanSE tool (7) was used to find tRNA genes, whereas rRNA genes were found by searches against models of the rRNA genes built from SILVA (8). Other noncoding RNAs, such as the RNA components of the protein secretion complex and the RNase P, were identified by searching the genome for the corresponding Rfam profiles using Infernal (http://infernal.janelia.org).

The ﬁnal draft assembly contained 44 contigs in 41 scaffolds, totaling 2.6 Mb with an *N*_50_ contig size of 148.6 kb. The largest contig was 245.9 kb with a G+C content of 42.0 mol%. The draft genome sequence has 2,668 CDSs, 61 tRNAs, 16 rRNAs, and two clustered regularly interspaced short palindromic repeats.

### Accession number(s).

This whole-genome shotgun project has been deposited at DDBJ/EMBL/GenBank under the accession number LIOK00000000. The version described in this paper is version LIOK01000000.
